# Optimizing a Dose Prescription as the First Step of Green Continuous Kidney Replacement Therapy

**DOI:** 10.1016/j.xkme.2025.101088

**Published:** 2025-08-16

**Authors:** Harin Rhee, Taeil Kim, Kyung Sook Jung, Eun Young Ku

**Affiliations:** 1 Department of Internal Medicine, Pusan National University School of Medicine, Busan, Republic of Korea; 2 Department of Nephrology, Biomedical Research Institute, Pusan National University Hospital, Busan, Republic of Korea; 3 CKRT specialized team, Pusan National University Hospital, Busan, Republic of Korea; 4 Hadan Jeil Internal Medicine Clinic, Busan, Republic of Korea

**Keywords:** Continuous renal replacement therapy, Continuous kidney replacement therapy, dialysis dose, green dialysis, green ICU, green nephrology, guideline implementation, intensive care unit, sustainable health care

## Abstract

**Rationale & Objective:**

Continuous kidney replacement therapy (CKRT) requires a large amount of fluid; however, it is often overused in clinical settings, leading to fluid waste. This study aimed to investigate the influence of dose optimization on fluid consumption and dialysis efficacy.

**Study Design:**

Single-center prospective study.

**Setting & Participants:**

All patients treated with CKRT at Pusan National University Hospital between May 1 and December 31, 2023.

**Quality Improvement Activities:**

The CKRT prescription dose was adjusted from the previous 35-25-30 mL/kg/h, targeting delivered doses between 20 and 25 mL/kg/h, as per the Kidney Disease: Improving Global Outcomes recommendations.

**Outcomes:**

The primary outcome was the change in total fluid consumption, and the secondary outcome was the differences in the pattern of biochemical parameter changes after 48 hours of CKRT before and after the study (serum urea nitrogen, creatinine, potassium, phosphate, and bicarbonate).

**Analytic Approach:**

General linear model for the primary outcome; repeated measures analysis of variance for the secondary outcome.

**Results:**

A total of 441 patients were included before (N = 210) and after (N = 231) participating in the study. The median age was 70.0 (61.0-77.5) years; 67.3% were male, and 17.6% had end-stage kidney disease. Before the study, the median prescribed dose for CKRT was 34.1 (33.0-35.4) mL/kg/h, with the median amount of fluid consumption being 53.6 (43.0-63.2) L/person/d. During the study periods, the median delivered dose was reduced to 27.4 mL/kg/h (*P* < 0.001), and total fluid consumption was reduced by 6.7 (3.7-9.8) L/person/d, with a median reduction in plastic package usage of 1.3 (0.8-1.9) (*P* < 0.001). The degree of biochemical changes was not significantly different before and after the study.

**Limitations:**

The fluid-saving effect may be greater in countries with heavier patients.

**Conclusions:**

Efforts to optimize CKRT dosing allowed for a 12.7% reduction in fluid consumption (ie, 6.7 L/person/d), without any additional changes in treatment efficacy.

## Plain Language Summary

Greening of intensive care is essential; however, it is often challenging in critically ill patients, because the greening process should not compromise patient safety. Continuous kidney replacement therapy is the most common method of dialysis in the intensive care unit, requiring large amounts of fluid. This quality improvement study controlled the delivered dialysis dose by reducing treatment intensity and analyzed its impact on fluid savings and treatment efficacy. Reducing the dose prescription by 3.3 mL/kg/h resulted in savings of approximately 6.7 L of fluid and 1.3 dialysate packages per person per day, without compromising dialysis efficacy. Although these are small changes at the individual patient level, their impact on the environment would be substantial if similar fluid-saving strategies were adopted in centers worldwide.

Environmental change and kidney care have a bidirectional relationship.^1^^,^^2^ Extreme heat caused by climate change can cause repetitive episodes of acute kidney injury via dehydration and may lead to chronic kidney disease, whereas kidney care practices, including hemodialysis or peritoneal dialysis, carry enormous environmental impacts, because they require high amounts of energy, water, and waste.^3^, ^4^, ^5^ Recently, an increasing number of nephrology societies have started implementing green nephrology during dialysis, with a water-saving strategy being central in this setting.^6^^,^^7^

Continuous kidney replacement therapy (CKRT) is one of the most widely used hemodialysis methods in intensive care units (ICUs). Owing to its continuous nature and slow removal of body fluids, CKRT is often used in patients with hemodynamic instability. In South Korea, the utility of CKRT in the ICU is increasing annually, and in 2016, approximately 80% of dialysis procedures performed in the ICU were CKRT.^8^

CKRT requires a considerable amount of fluid, which is largely dependent on the physician’s prescription of the targeted dose of treatment. Based on the Randomized Evaluation of Normal vs. Augmented Level of Renal Replacement Therapy (RENAL) or Acute Renal Failure Trial Network (ATN) trials, which showed no additional benefit with doses higher than 20-25 mL/kg/h, the 2012 Kidney Disease: Improving Global Outcomes (KDIGO) guideline recommends the CRRT dose to be prescribed to deliver this amount of dialysis with evidence level 1A.^9^, ^10^, ^11^ However, in clinical practice, the actually delivered dose often remains unmeasured, and a prescription dose is usually higher than required.^12^, ^13^, ^14^, ^15^

Although a prescription higher than the recommended dose may not add additional harm or benefit to patient outcomes, it does harm the environment by resulting in the waste of a considerable amount of fluid.^9^^,^^10^ We hypothesized that efforts to optimize the CKRT dose may have beneficial effects on the environment without compromising treatment efficacy.

This study aimed to investigate the influence of dose optimization on fluid consumption during CKRT and its impact on treatment efficacy.

## Methods

### Baseline Practice Pattern Assessment

We retrospectively reviewed patients’ medical charts from September 1 to April 30, 2023. The prescribed and delivered doses were reviewed, and the treatment downtime (hours off CKRT/24 h) at our institute was calculated.^16^

### Study Design and Population

This is a single-center prospective study conducted at the third affiliated hospital in South Korea. During the study period, the CKRT prescription dose was reduced based on the previous 8 months of treatment patterns, targeting delivered doses between 20 and 25 mL/kg/h. We included all patients receiving CKRT for >24 h, including patients with end-stage kidney disease previously receiving maintenance dialysis. Approval to perform the analyses was obtained from the Pusan National University IRB Committee (approval number: 2404-010-138), which waived the requirement for informed consent.

### Quality Improvement Intervention

ICU nurses and a specialized CKRT team, consisting of 2 nurses and 2 nephrology staff members, were engaged in this dose optimization program. The CKRT prescription dose was reduced from 35 mL/kg/h to 25-30 mL/kg/h. Doses were calculated based on the premorbid body weight or body weight at hospital admission (if no premorbid body weight information was available). During the daytime, the specialized CKRT team adjusted the dialysate and replacement flow rates, and at night, ICU nurses controlled the flow rate every 8 hours. The actual delivered dose was the mean of the effluent flow rate, which was checked and recorded every 8 hours.

CKRT prescription and source of fluid consumption during CKRT

A detailed description of the CKRT surgery has been described in our previous study.^14^^,^^17^ In brief, CKRT was performed using the pre- and postdilution continuous venovenous hemodiafiltration mode. Before the study, the usual prescription dose was 35 mL/kg/h with hemodialysis and hemofiltration at a 1:1 ratio, which was lowered to 25-30 mL/kg/h during the study period. Heparin was used as the primary anticoagulant; however, in cases of bleeding tendency, nafamostat mesylate was used as an alternative.^14^

A major source of fluid consumption during CKRT is the dialysate (5-L bag) and replacement solution (5-L bag). In continuous venovenous hemodiafiltration, with hemodialysis and hemofiltration at a 1:1 ratio, a prescription dose of 30 mL/kg/h for a 70-kg patient typically requires 2.1 L of fluid/h, which corresponds to 50.4 L of fluid/day. During the machine priming process, approximately 1.5 L of normal saline is required; however, this amount of fluid was not included in the calculation of fluid usage in this study.

### Data Collection and Definitions

The parameters included demographics, comorbid conditions, disease severity, and quantity of dialysate or replacement solution consumed for CKRT. Comorbid conditions included diabetes, hypertension, chronic kidney disease, cancer, and end-stage kidney disease, for which information was obtained from physician notes. Disease severity at the time of CKRT was assessed using the sequential organ failure assessment score, and information on sepsis was obtained based on the physician’s note. Data on the number of dialysate or replacement solution packages consumed were retrospectively extracted from the electronic medical record claim database. The total quantity of fluid consumed was estimated by multiplying the number of consumed packages by 5 L, because each bag contains this amount of fluid.

The total fluid consumption per person per day during CKRT was calculated for each patient as follows: total quantity of fluid used during CKRT (L) divided by the number of days in which CKRT was performed. The volume of normal saline used in the CKRT priming process was excluded from the calculations.

### Outcome Measurements

The primary outcome was the change in total fluid consumption per person per day before and after the study. The secondary outcomes included changes in serum levels of biochemical parameters after 48 hours of CKRT, before and after the study. These parameters included serum urea nitrogen, creatinine, potassium, phosphate, and bicarbonate levels.

### Statistical Analysis

Normality of the data was verified using the Kolmogorov–Smirnov test. Continuous variables were expressed as median and interquartile range (IQR) or mean± standard deviation, as appropriate. Differences in the parameters before and after the study were compared using *t* test or Kruskal–Wallis test. Categorical variables are summarized as numbers and percentages and compared using the χ^2^ test. Changes in total fluid consumption before and after the study were tested using a general linear model adjusted for age, diabetes, hypertension, cancer, sepsis, sequential organ failure assessment score, and serum levels of albumin, hemoglobin, serum urea nitrogen, creatinine, and potassium. Changes in biochemical parameters were compared using a repeated measures analysis of variance test. A 2-sided *P* value < 0.05 was deemed statistically significant. We used IBM SPSS Statistics for Windows (version 29.0; SPSS Inc Corp).

## Results

### Baseline CKRT Practice Pattern in Our Institute

The baseline CKRT practice pattern at our institute was assessed between September 1, 2022 and April 30, 2023. In 210 patients who met the inclusion criteria for the assessment of baseline CKRT practice, the median of prescription and delivered dose was 34.1 (IQR, 33.0-35.4) and 31.3 (IQR, 29.5-32.9) mL/kg/h respectively, with a median delivery rate of 92.8% (IQR, 87.8%-95.3%). Targeting an actual delivered dose between 20-25 mL/kg/h, we reduced the prescription dose to 25-30 mL/kg/h starting on May 1, 2023.

### Changes in CKRT Doses Before and After the Program

After the study, the median prescribed and delivered CKRT doses were 29.7 (27.5-33.1) mL/kg/h and 27.3 (24.9-29.5) mL/kg/h, respectively, demonstrating a reduction of 3.3 (2.3-4.3) mL/kg/h from baseline. Before the program, only 3.3% of patients were received a CKRT dose between 20 and 25 mL/kg/h, and >70% of patients received a dose >30 mL/kg/h. After the study, the proportion of patients receiving 20-25 mL/kg/h increased to 23.4%, whereas the proportion of patients receiving a dose >30 mL/kg/h decreased to 20.8% (Table S1).

### Baseline Patient Characteristics After the Quality Improvement Program

From May 1 to December 31, 2023, the data of 231 patients were collected. The median patient age was 71.0 (IQR, 62.0-75.0) years; 64.9% were male, 55.8% had diabetes, 19.3% had cancer, and 19.0% had end-stage kidney disease. At the time of CKRT initiation, the mean sequential organ failure assessment score was 10.76 ± 3.76, and 30.6% had sepsis. These baseline characteristics did not differ before and after the study period, except for the lower prevalence of cancer after the program. Details are summarized in Table 1.

**Table 1:** Baseline Characteristics of Patients Before and After CKRT Dose Optimization

	Total (N = 441)	Before CKRT dose optimization (N = 210)	After CKRT dose optimization (N = 231)	*P* Value
Demographics				
Age, y	70.0 (61.0-77.5)	68.0 (60.0-76.0)	71.0 (62.0-78.0)	0.064
Male, %	297 (67.3)	147 (70.0)	150 (64.9)	0.257
Weight, kg	63.91 ± 14.11	64.68 ± 13.90	63.22 ± 14.28	0.297
BMI, kg/m^2^	23.76 ± 6.64	23.62 ± 4.11	23.89 ± 8.23	0.673
Comorbid conditions				
Diabetes, n (%)	228 (511.7)	99 (47.1)	129 (55.8)	0.068
Hypertension, n (%)	251 (56.9)	110 (52.4)	141 (61.0)	0.067
ESKD, n (%)	76 (17.6)	33 (16.0)	43 (19.0)	0.412
Cancer, n (%)	105 (24.2)	61 (29.6)	44 (19.3)	0.012
Disease severity				
Sepsis, %	145 (33.2)	75 (36.1)	70 (30.6)	0.224
Anuria, %	281 (65.2)	137 (66.2)	144 (64.3)	0.679
MAP, mm Hg	78.69 ± 17.03	76.67 ± 17.52	78.71 ± 16.61	0.980
Vasopressors, %	287 (65.1)	134 (63.8)	153 (66.2)	0.594
Ventilator, %	263 (59.6)	123 (58.6)	140 (60.6)	0.664
SOFA score	10.78 ± 3.85	10.80 ± 3.96	10.76 ± 3.76	0.915
Laboratory test at CKRT initiation				
WBC, 10^3^/μL	14.96 ± 22.72	15.18 ± 30.25	14.75 ± 12.51	0.842
Platelet, 10^3^/μL	144.63 ± 105.47	139.15 ± 112.46	149.59 ± 98.67	0.305
Hemoglobin, g/dL	9.80 ± 2.37	9.67 ± 2.27	9.93 ± 2.45	0.264
Total protein, g/dL	5.38 ± 1.27	5.27 ± 1.30	5.48 ± 1.24	0.088
Albumin, g/dL	2.99 ± 0.73	2.93 ± 0.72	3.07 ± 0.74	0.038
SUN, mg/dL	58.09 ± 32.31	56.95 ± 32.21	59.13 ± 32.43	0.482
Creatinine, mg/dL	4.21 ± 3.16	4.16 ± 2.93	4.24 ± 3.35	0.789
Sodium, mEq/L	136.17 ± 7.30	136.74 ± 6.76	135.66 ± 7.75	0.123
Potassium, mEq/L	4.73 ± 1.07	4.68 ± 1.01	4.77 ± 1.16	0.365
PT, INR	1.67 ± 0.75	1.68 ± 0.60	1.66 ± 0.86	0.832
CKRT surgery				
Filter life, h	22.0 (10-0-40.1)	21.9 (12.9-34.0)	24.5 (6.8-68.5)	0.095
Prescribed dose, mL/kg/h	32.9 (29.4-34.8)	34.1 (33.0-35.4)	29.7 (27.5-33.1)	<0.001
Delivered dose, mL/kg/h	29.3 (26.3-31.9)	31.3 (29.5-32.9)	27.3 (24.9-29.5)	<0.001
Dose delivery rate, %	91.7 (87.2-95.2)	92.8 (87.8-95.3)	92.1 (86.8-95.6)	0.717
Downtime, %/24 h	2.52 (0.00-1.59)	2.76 (0.00-4.77)	2.38 (0.00-4.39)	0.413
CKRT duration, d	6.24 ± 6.65	6.77 ± 7.61	5.77 ± 5.62	0.120

Abbreviations: CKRT, continuous kidney replacement therapy; BMI, body mass index; ESKD, end-stage kidney disease; MAP, mean arterial pressure; SOFA, sequential organ failure assessment; WBC, white blood cell; SUN, serum urea nitrogen; PT, prothrombin time.

### Changes in Total Fluid Consumption Before and After Dose Optimization

**Figure 1:**
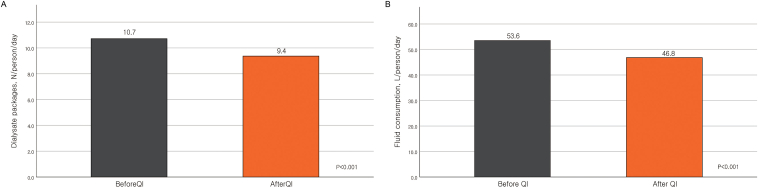
Changes in the number of dialysate packages (A) and total fluid consumption (B) before and after QI program. Abbreviation: QI, quality improvement.

The median total fluid consumption during CKRT was 53.6 (43.0-63.2) L/d at baseline, which was reduced to 46.8 (37.8–56.0) L/d, resulting in a fluid saving of 6.7 (3.7-9.8) L/person/d. The consumption of dialysate packages was reduced from 10.7 (8.6-12.6) to 9.4 (7.5-11.2) bags/person/d, a reduction of 1.3 (0.7-1.9) bags/d (Fig 1). These fluid- and package-saving effects remained significant in the general linear model, adjusted for multiple factors, including age, sepsis, cancer, and serum levels of serum urea nitrogen, creatinine, and potassium at CKRT initiation (Table 2).

**Table 2:** Changes in Fluid Consumption Before and After CKRT Dose Optimization

Univariable Model	Before	After	Mean Difference	P Value
Total fluid consumption/person/d	53.6 (43.0-63.2)	46.8 (37.5-56.0)	6.74 (3.72-9.76)	<0.001
Total package consumption/person/d	10.7 (8.6-12.6)	9.4 (7.5-11.2)	1.35 (0.75-1.95)	<0.001
Multivariable Model				
Total fluid consumption/person/d	53.6 (43.0-63.2)	46.8 (37.5-56.0)	6.93 (3.79-10.07)	<0.001
Sepsis, yes			-6.03 (-12.35-0.28)	0.061
Cancer, yes			-0.191 (-3.92-3.53)	0.920
Age, y			-0.09 (-0.20-0.02)	0.118
SUN at CKRT initiation			-0.00 (-0.06-0.05)	0.978
sCr at CKRT initiation			-0.46 (-1.02-0.10)	0.107
sK at CKRT initiation			0.78 (-0.70-2.26)	0.299
Total package consumption/person/d	10.7 (8.6-12.6)	9.4 (7.5-11.2)	1.39 (0.76-2.02)	<0.001
Sepsis, yes			-1.21 (-0.04-0.01)	0.060
Cancer, yes			-0.04 (-0.78-0.71)	0.918
Age, y			-0.02 (-0.04-0.01)	0.118
SUN at CKRT initiation			0.00 (-0.01-0.01)	0.979
sCr at CKRT initiation			-0.09 (-0.21-0.02)	0.105
sK at CKRT initiation			0.16 (-0.14-0.45)	0.298

Abbreviations: CKRT, continuous kidney replacement therapy; SUN, serum urea nitrogen; sCr, serum creatinine; sK, serum potassium.

### Changes in Biochemical Parameters Before and After the Study

**Figure 2:**
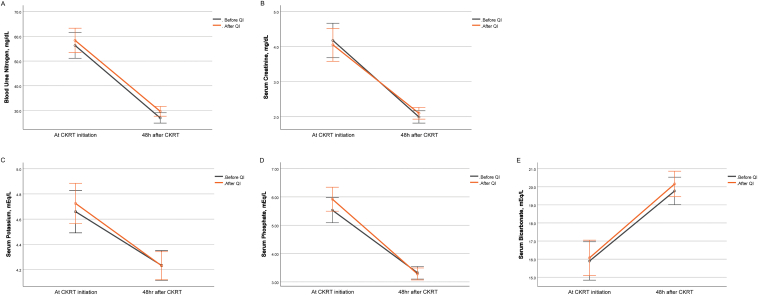
Changes in blood levels of biochemical parameters before and after the study: serum urea nitrogen (A), serum creatinine (B), serum potassium (C), serum phosphate (D), and serum bicarbonate (E) levels.

As shown in Fig 2, initial blood levels of serum urea nitrogen, creatinine, potassium, and phosphate were significantly decreased, whereas serum bicarbonate levels were significantly elevated after 48 hours of CKRT. However, there were no significant differences in the degrees of these changes before and after the study (Table S2).

## Discussion

This study evaluated the influence of CKRT dose optimization on fluid consumption and treatment efficacy. At our center, the CKRT prescription was higher than the KDIGO recommendations, and reducing the CKRT dose prescription by 3 mL/kg/h for each patient resulted in a 6.7 L reduction in daily fluid consumption and a reduction of 1.3 plastic packages, with no adverse effect treated with dialysis efficacy.^11^ Because the mean CKRT duration at our center is 6.2 days and approximately 350 patients receive CKRT annually, the reduction in fluid consumption of 6.7 L/person/d corresponds to a savings of 14,539 L of fluid and 2,821 dialysate packages per year. If this strategy is adopted by more centers worldwide, an enormous amount of fluid could be saved each year. The novelty of this study is that to our knowledge, it is the first to suggest the influence of the CKRT practice pattern changes on environmentally sustainable development during the care of critically ill patients admitted to the ICU.

CKRT requires a considerable amount of fluid. If a patient weighing 70 kg received CKRT with a usual prescription dose of 30 mL/kg/h, the arithmetically required dialysate and/or replacement solution for one day is approximately 50 L (30 mL/kg/h × 70 kg × 24 h). Because the dialysate and replacement solutions are highly purified water, the production of these solutions requires a much higher amount of water. Moreover, this fluid is packed in plastic bags weighing approximately 62 g per bag. Because each bag typically contains 5 L of fluid, 10 plastic bags are required to hold 50 L of solution, which corresponds to 620 g of plastic. Although the water footprint of plastic varies by type and manufacturing technique, creating 1 kg of plastic typically requires approximately 180 L of water, and 620 g of plastic is equivalent to 111.6 L of water.^2^^,^^3^ Therefore, it is estimated that at least 161.6 L of water is required per person per day for CKRT. Because we saved 6.7 L of fluid and its packaging (1.3 bags of dialysate; approximately 80.6 grams of plastic, which corresponds to 14.5 L of water) during the study period, the estimated amount of water saved is 21.2 L/person/d.

KDIGO recommends a CKRT dose to be delivered at 20-25 mL/kg/h with evidence level 1A because the previous 2 randomized controlled trials proved that a higher than these doses does not affect patient mortality.^9^, ^10^, ^11^ However, many centers around the world prescribe a much higher dose than recommended or even do not target a specific dose unless educated.^13^^,^^15^^,^^17^^,^^18^ A nationwide survey in the US indicated that <50% of practitioners target a specific dose, and only 15% of patients have regular dose assessment during dialysis.^12^ Although the survey was only asked for intermittent hemodialysis cases, practice patterns on dose prescription may not differ for CKRT. Based on the direct effect of dose optimization on water saving and conservation of the environment, routine measurement and monitoring of CKRT doses need to be established as the standard of care. Recently, 3 randomized controlled trials comparing patient and kidney outcomes between standard and lower-than-standard doses are recruiting participants: the Kidney Evaluation of Therapy at ZEro vs REduced Intensity trial (standard vs 10-15 mL/kg/h, NCT06021288); the Lower Intensity of Maintenance Intensity Trial (standard vs 12 mL/kg/h, NCT06014801); the Withholding Intensity in Severe Disease Or Maintenance trial (standard vs 10-15 mL/kg/h, NCT06446739). These trials may provide further opportunities to save even more fluid during CKRT.

This study had some limitations. First, we were unable to measure the water footprint because the exact amount of water required to produce one bag of dialysate or replacement solution was not disclosed by the medical industry. Second, because the median CKRT dose delivered after the study was 27.3 mL/kg/h, which was higher than the KDIGO recommendation, the fluid-saving effect was suboptimal. We are currently lowering the prescription dose to 23-25 mL/kg/h and expect a doubled fluid-saving effect. Third, we did not measure the additional beneficial effects of CKRT dose reduction, such as reduced clearance of antibiotics during CKRT.^19^ However, this does not affect changes in fluid usage, which is our primary endpoint.

In conclusion, we demonstrated the influence of a dose optimization strategy on fluid conservation. Reducing dose prescription by 3.3 mL/kg/h resulted in savings of approximately 6.7 L of fluid and 1.3 dialysate packages per person per day, without further harm to dialysis efficacy. Although there have been small changes in clinical practice, their impact on the environment is enormous. We hope that similar fluid-saving strategies will be adopted in other centers worldwide, which could be the first step toward green CKRT.
