# Supplementary material for: Optimizing a Dose Prescription as the First Step of Green Continuous Kidney Replacement Therapy
Source: Kidney Med. 2025 Aug 16;7(10):101088. doi: 10.1016/j.xkme.2025.101088 (PMC12495464; doi:10.1016/j.xkme.2025.101088)
Supplement: Supplementary File (PDF) — Tables S1-S2. [file mmc1.pdf]

Table S1. Changes in prescription and delivered dose before and after the program

	Prescription dose		Delivered dose	
	Before QI N=210	After QI N=231	Before QI N=210	After QI N=231
<20mL/kg/h	0	0	3(1.4%)	8(3.5%)
20-25mL/kg/h	2(1.0%)	15(6.5%)	7(3.3%)	54(23.4%)
25.1-30 mL/kg/h	10(4.8%)	108(46.7%)	52(24.8%)	121(52.4%)
>30mL/kg/h	198(94.2%)	108(46.7%)	148(70.5%)	48(20.8%)

Abbreviations: QI, quality improvement

Table S2. Changes in biochemical parameters before and after the QI program

	Before QI		After QI		P-value
	At CKRT initiation	After 48hr of CKRT	At CKRT initiation	After 48hr of CKRT	
Blood Urea Nitrogen, mg/dL	56.28±33.08	27.02±12.97	58.33±32.12	29.71±13.14	0.868
Creatinine, mg/dL	4.17±3.07	1.99±1.08	4.05±3.12	2.09±1.12	0.526
Potassium, mEq/L	4.65±0.99	4.23±0.76	4.72±1.12	4.23±0.72	0.625
Phosphate, mEq/L	5.53±2.62	3.32±1.59	5.92±2.94	3.28±1.20	0.231
Bicarbonate, mEq/L	15.89±5.67	19.77±4.36	16.07±5.37	20.16±3.58	0.802

Abbreviations: QI, quality improvement; CKRT, continuous kidney replacement therapy
